# Efficacy and Safety of TROP-2-Targeting Antibody–Drug Conjugate Treatment in Previously Treated Patients with Advanced Non-Small Cell Lung Cancer: A Systematic Review and Pooled Analysis of Reconstructed Patient Data

**DOI:** 10.3390/cancers17111750

**Published:** 2025-05-23

**Authors:** Sara Stumpo, Andrea Carlini, Francesco Mantuano, Alessandro Di Federico, Barbara Melotti, Francesca Sperandi, Valentina Favorito, Andrea De Giglio

**Affiliations:** 1 Department of Medical and Surgical Sciences (DIMEC), Alma Mater Studiorum—University of Bologna, Via Zamboni 33, 40126 Bologna, Italy; andrea.carlini10@studio.unibo.it (A.C.); francesco.mantuano3@studio.unibo.it (F.M.); alessandr.difederic2@unibo.it (A.D.F.); valentina.favorito@studio.unibo.it (V.F.); andrea.degiglio2@unibo.it (A.D.G.); 2 Medical Oncology Unit, IRCCS Azienda Ospedaliero—Universitaria di Bologna, Via P. Albertoni 15, 40138 Bologna, Italy; barbara.melotti@aosp.bo.it (B.M.); francesca.sperandi@aosp.bo.it (F.S.)

**Keywords:** non-small cell lung cancer, antibody–drug conjugates, TROP2, sacituzumab, datopotamab

## Abstract

Background: Docetaxel is the standard of care for advanced non-small cell lung cancer (NSCLC) after platinum-based chemotherapy and/or immunotherapy but is associated with modest clinical outcomes and considerable toxicity. Sacituzumab govitecan and datopotamab deruxtecan are trophoblast cell surface antigen (TROP)-2-directed antibody–drug conjugates (ADCs) that showed encouraging activity in pretreated patients with advanced NSCLC. This systematic review and pooled analysis aims to comprehensively assess the efficacy and safety of anti-TROP-2 ADCs compared to docetaxel in pretreated patients with advanced NSCLC. Methods: A systematic search through PubMed and EMBASE before 31 January 2025 was performed to identify eligible studies. Randomized controlled phase III trials comparing an anti-TROP-2 regimen to docetaxel in patients with pretreated advanced NSCLC were included. Overall survival (OS), progression-free survival (PFS), and grade ≥ 3 treatment-related adverse events (TRAEs) were extracted from the identified trials. A pooled analysis of reconstructed patient data and meta-analysis employing the random-effect model were used to summarize the efficacy and safety outcomes. Results: Across the two trials included, 1207 patients were enrolled, 598 in the TROP-2 ADC arm and 609 in the docetaxel arm. Anti-TROP-2 treatment did not produce significant improvements in OS (HR: 0.90; 95% CI, 0.78–1.03; P = 0.13) and PFS (HR: 0.84; 95% CI, 0.68–1.02; P = 0.08), compared to docetaxel, even in patients with a nonsquamous histology (OS HR: 0.86; 95% CI, 0.73–1.01; P = 0.06; PFS HR: 0.76; 95% CI, 0.52–1.12; P = 0.17). Across the subgroup analyses, a statistically significant improvement in OS was observed in patients with actionable genomic alterations (AGAs) (HR: 0.63; 95% CI, 0.41–0.95; P = 0.03). Compared to docetaxel, the anti-TROP-2 regimen demonstrated a lower risk of developing grade ≥ 3 TRAEs (RR: 0.76; 95% CI, 0.55–1.05; P = 0.09). Conclusions: The anti-TROP-2 regimen showed a better safety profile but failed to demonstrate a relevant clinical improvement over docetaxel. Anti-TROP-2 ADCs could find a role in the management of patients with AGAs.

## Simple Summary

This pooled analysis aims to assess the survival and safety outcomes of the trophoblast cell-surface antigen 2-directed antibody–drug conjugates (ADCs) sacituzumab govitecan and datopotamab deruxtecan compared to docetaxel in pre-treated patients with advanced/metastatic non-small cell lung cancer by integrating data from the randomized phase III trials EVOKE-01 and TROPION-Lung01. According to our findings, anti-TROP-2 ADCs did not produce significant improvements in overall survival (hazard ratio [HR]: 0.90; P = 0.13) and progression-free survival (HR: 0.84; P = 0.08) compared to docetaxel, regardless of histology. A significant improvement in overall survival was observed in patients with actionable genomic alterations (AGAs) (HR: 0.63; 95% CI, 0.41–0.95; P = 0.03). Treatment with ADCs targeting TROP-2 did not show clinical benefit compared to the standard of care in pretreated patients with NSCLC but could find a role in the management of patients with AGAs. The absence of a clear correlation between membrane TROP-2 expression and clinical outcomes underscores the urgent need for robust predictive biomarkers.

## 1. Introduction

Non-small cell lung cancer (NSCLC) represents a significant global health burden, accounting for approximately 80–85% of lung cancer diagnoses [1]. The advent of immune checkpoint inhibitors (ICIs) and targeted therapies directed at oncogenic drivers has fundamentally transformed the treatment landscape for advanced or metastatic NSCLC [2,3]. Nevertheless, a substantial proportion of patients experience disease progression, after which therapeutic options become severely limited. Currently, docetaxel-based chemotherapy is the standard treatment following platinum-based chemotherapy and/or immunotherapy [4], though it offers only modest efficacy, with a median overall survival (mOS) of 10 to 12 months and significant toxicity [4,5,6,7,8].

Emerging therapeutic strategies are focused on targeting the trophoblast cell surface antigen 2 (TROP-2), a cell membrane glycoprotein receptor that plays a critical role in promoting tumor growth, proliferation, and metastasis by acting as a transmembrane transducer of intracellular calcium signaling [9]. TROP-2 expression is upregulated in many solid tumors, which has led in recent years to increasing interest in this membrane receptor and the subsequent development of targeted therapies [10]. Sacituzumab govitecan (SG) and datopotamab deruxtecan (Dato-DXd) are antibody–drug conjugates (ADCs) composed of an anti-TROP-2 antibody linked to a topoisomerase I inhibitor: SN-38 (the active metabolite of irinotecan) and deruxtecan, respectively [11,12]. Anti-TROP-2 antibody binds to TROP-2-expressing tumor cells and allows the selective delivery of cytotoxic agents, thereby enhancing therapeutic efficacy while minimizing off-target effects [13]. However, the presence of enzymes within the tumor microenvironment that facilitate the cleavage of the linker between the antibody and the drug contributes to extracellular payload release [14].

These agents have demonstrated encouraging activity in breast cancer [15], leading to SG’s approval in this setting, and promising results in early-phase studies of pretreated patients with NSCLC [16,17].

Promising results have also been reported for sacituzumab tirumotecan, an ADC targeting TROP-2 that employs a belotecan-derived topoisomerase I inhibitor as its cytotoxic payload. Sacituzumab tirumotecan was recently approved in China for the treatment of pretreated patients with triple-negative breast cancer, based on the positive outcomes of the phase III OptiTROP-Breast01 trial [18]. The drug has also demonstrated promising antitumor activity in patients with advanced urothelial carcinoma [19].

Recently, two randomized phase III trials, EVOKE-01 and TROPION-Lung01, evaluated the clinical efficacy of SG and Dato-DXd in patients with previously treated advanced or metastatic NSCLC compared to single-agent docetaxel [20,21]. While neither study demonstrated a statistically significant improvement in mOS, TROPION-Lung01 reported a modest but statistically significant improvement in median progression-free survival (mPFS). Additionally, both therapies exhibited variability in subgroup analyses, particularly with respect to tumor histology and the presence of actionable genomic alterations (AGAs). The discrepancies observed in outcomes across studies highlight the need for a comprehensive evaluation to better define the therapeutic potential of anti-TROP-2 ADCs.

This systematic review and pooled analysis aims to synthesize evidence from randomized controlled trials (RCTs) enrolling patients with advanced NSCLC to assess the safety and efficacy of anti-TROP-2 ADCs compared to docetaxel. By providing integrated insights, we aim to clarify the clinical utility of these agents and their potential to improve outcomes for this challenging patient population.

## 2. Materials and Methods

### 2.1. Search Strategy and Selection Criteria

Studies published before 31 January 2025, were searched in MEDLINE (PubMed) and EMBASE using the following terms: “(non-small cell lung cancer or NSCLC or non-small-cell lung cancer) and (TROP2 or TROP-2 or sacituzumab or datopotamab) and (clinical trial or trial or phase III or phase 3)”. The inclusion criteria were (1) phase III RCTs enrolling patients with pretreated advanced or metastatic NSCLC; (2) a comparison of TROP-2-targeting ADCs treatment vs. docetaxel as the control arm, and (3) the availability of hazard ratios (HRs) and relative 95% confidence intervals (CIs) for PFS and OS. All articles were screened for relevance based on the title and abstract by two different authors (S.S. and A.D.G.), and relevant studies were fully evaluated to assess eligibility for inclusion.

Abstracts from major international conferences of the last 5 years were also reviewed.

**Figure 1:**
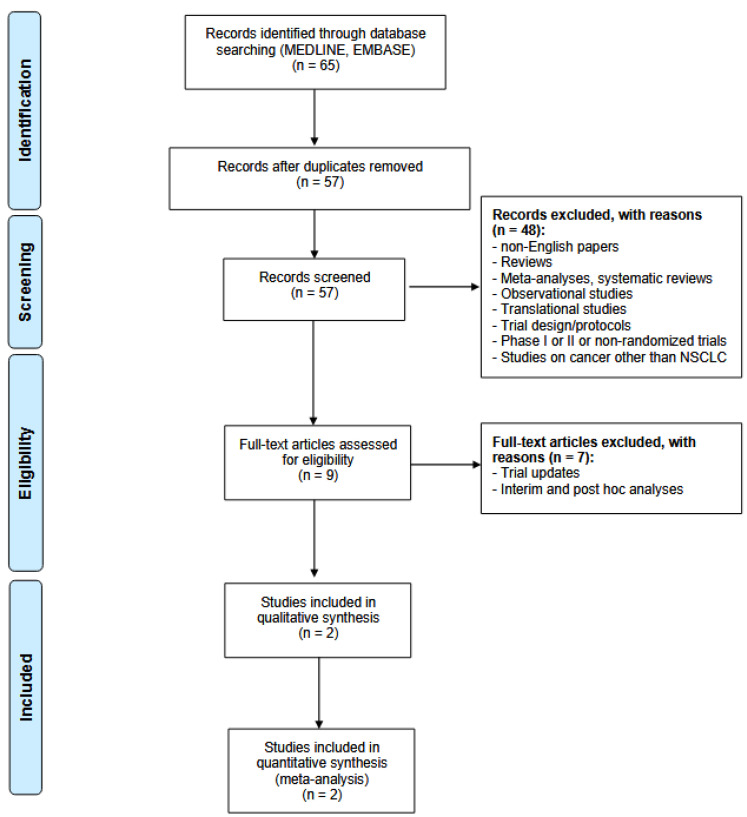
Preferred Reporting Items for Systematic Reviews and Meta-Analyses (PRISMA) flow-chart.

This systematic review and pooled analysis followed the Preferred Reporting Items for Systematic Reviews and Meta-analyses (PRISMA) reporting guideline (Figure 1) [22] and was registered in PROSPERO (CRD420251054495).

### 2.2. Risk of Bias

The risk of bias for each trial was independently assessed by two authors (S.S. and A.D.G.) using the Cochrane risk of bias tool for randomized trials version 2 (Supplemental Material Table S1). The results of the current work were interpreted according to the risk of bias, and any disagreement was resolved through discussion to reach a consensus.

### 2.3. Outcome Measures

Outcomes of interest, including efficacy (OS and PFS) and safety (treatment-related adverse events, TRAEs), were extracted from the included RCTs. OS was defined as the time from treatment assignment to death from of any cause. PFS was defined as the time from treatment assignment to disease progression or death from any cause. Grade ≥ 3 TRAEs were defined according to the Common Terminology Criteria for Adverse Events [23].

### 2.4. Data Extraction and Statistical Analysis

HRs and 95% CIs for OS and PFS were extracted from each study. HRs and CIs of subgroups stratified by sex, age, histology, and AGAs were also extracted. Since in TROPION-Lung01, CIs were not reported in the subgroup analysis forest plot, the WebPlotDigitizer v5 tool was used to extract the data. The numbers of patients developing a grade ≥ 3 TRAE in the experimental and control arms from each study were also extracted.

The meta-analysis was performed using Review Manager Version 5.4 software. HRs and relative 95% CIs were used to summarize time-to-event outcomes (OS and PFS). Risk ratios (RRs) and relative 95% CIs were used to describe grade ≥ 3 TRAEs. The inverse variance method was used for the HRs, while the Mantel–Haenszel method was used for the risk ratios (RRs). Statistical heterogeneity between studies was examined using the χ^2^ test and the I-squared (I^2^) statistic. A random-effect model was adopted for all the analyses.

Individual patient data (IPD) were extracted from Kaplan–Meier curves and then reconstructed using the table of numbers at risk at each time point with IPDfromKM Version 1.2.3.0 [24]. Every reconstructed Kaplan–Meier curve was carefully examined to ensure that it was consistent with the original Kaplan–Meier curves. Survival analyses were then performed in R studio 2024.09.1+394 using the Kaplan–Meier methodology. HRs and 95% CIs were estimated using Cox proportional hazards regression and P values were calculated with the log-rank test. All tests were 2-sided, CIs were 95%, and a P value  <  0.05 was considered significant.

## 3. Results

### 3.1. Systematic Research

The systematic search identified 65 articles, of which 57 non-duplicated records were screened by title and abstract. Nine studies were fully reviewed for eligibility. Two RCTs met the inclusion and exclusion criteria and were eventually included: the EVOKE-01 and TROPION-Lung01 trials (Figure 1).

Both studies included adult patients with stage III or IV NSCLC that progressed after platinum-based chemotherapy in combination or sequentially with an anti-PD-(L)1-containing regimen. Patients with AGAs were included (EGFR, ALK, ROS1, NTRK, BRAF, MET exon 14 skipping, or RET) and they all received at least one prior targeted therapy with tyrosine kinase inhibitors (TKIs) and platinum-based chemotherapy with or without anti-PD-(L)1 (in the EVOKE-01 trial all patients received anti-PD-(L)1 treatment).

All patients had to have an Eastern Cooperative Oncology Group (ECOG) performance status score of zero or one at screening, and patients with asymptomatic or previously treated brain metastases were eligible.

### 3.2. Population Characteristics

Across the EVOKE-01 and TROPION-Lung01 trials, a total of 1207 patients were enrolled. Overall, 593 patients received docetaxel (75 mg/m^2^ once every 3 weeks), and 595 patients received an ADC targeting TROP-2 (Dato-DXd 6 mg/kg once every 3 weeks or SG 10 mg/kg once on days 1 and 8 every 3 weeks). Nineteen patients randomly assigned to the EVOKE-01 trial were not treated.

Demographic and baseline characteristics were similar between groups (Table 1). The patients’ median age was 64.5 years (28.5–84) in the anti-TROP-2 arm and 64 years (28–85.5) in the docetaxel arm. Nonsquamous histology was the predominant subtype in both cohorts (73.4% vs. 73.6%). The PD-L1 status was predominantly ≥ 1% (56.9% vs. 52.9%) and AGAs were reported in 11.5% and 12.7% of patients in the anti-TROP-2 and docetaxel arms, respectively. Brain metastases were slightly more common in the docetaxel group (21.4% vs. 19.1%).

### 3.3. Efficacy and Subgroup Analyses

Across the EVOKE-01 and TROPION-Lung01 trials, 598 patients were randomly assigned to receive anti-TROP-2 ADC treatment (SG and Dato-DXd) and 609 patients to receive docetaxel.

**Figure 2:**
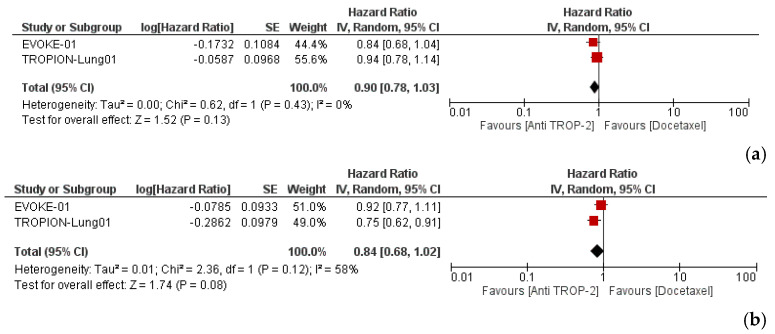
Efficacy of the TROP-2-targeting antibody–drug conjugate vs. docetaxel in pretreated patients with advanced or metastatic NSCLC. (**a**) Overall survival (OS); (**b**) progression-free survival (PFS).

Anti-TROP-2 treatment did not demonstrate a statistically significant improvement in OS (HR: 0.90; 95% CI, 0.78–1.03; P = 0.13) and PFS (HR: 0.84; 95% CI, 0.68–1.02; P = 0.08), compared to docetaxel (Figure 2a,b).

**Figure 3:**
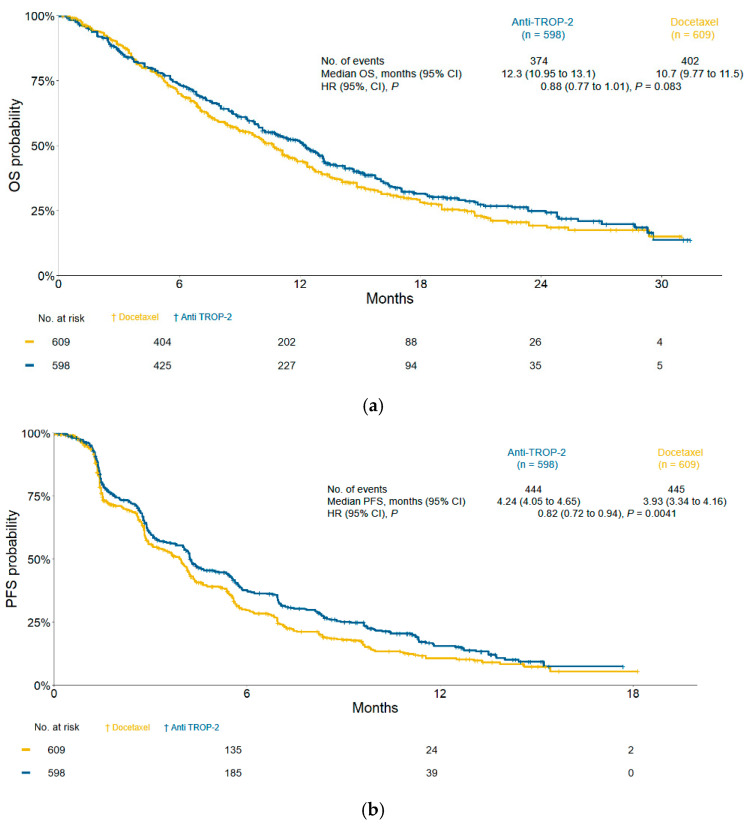
Survival outcomes of pretreated patients with advanced or metastatic NSCLC receiving the anti-TROP-2 regimen vs. docetaxel. (**a**) Overall survival (OS) and (**b**) progression-free survival (PFS) in the intention-to-treat population. HR: hazard ratio; CIs: confidence intervals. HRs and 95% CIs were calculated using the Cox proportional hazards model; P values were derived from the log-rank test.

IPD were reconstructed from the Kaplan–Meier curves of the two studies. Compared to docetaxel, the anti-TROP-2 regimen showed a statistically significant improvement in PFS, with an mPFS of 4.2 vs. 3.9 months (HR: 0.82; 95% CI 0.72–0.94; P = 0.0041), but not in OS (mOS 12.3 vs. 10.7 months; HR: 0.88; 95% CI 0.77–1.01; P = 0.083) (Figure 3a,b).

**Figure 4:**
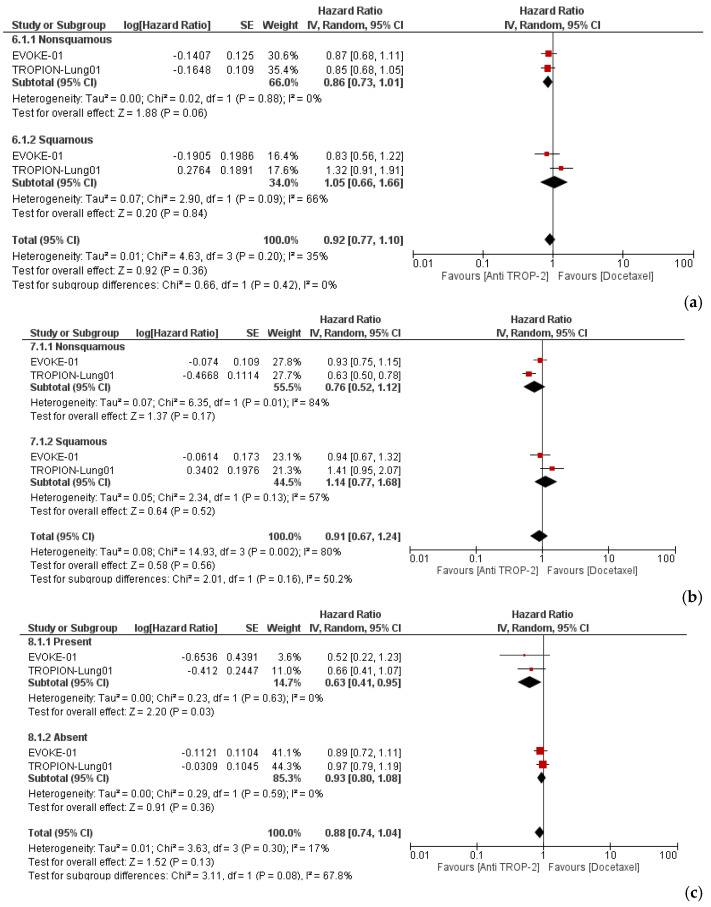

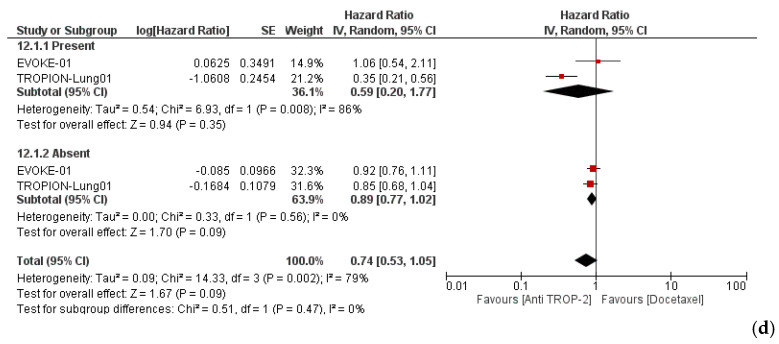
Forest plot of survival outcomes in the subgroup analyses. (**a**,**b**) Overall survival (OS) and progression-free survival (PFS) by histology: nonsquamous and squamous. (**c**,**d**) OS and PFS by actionable genomic alterations (AGAs): present and absent.

The PFS and OS HRs by sex, age (<65 or ≥65), histology (nonsquamous or squamous), and the presence of AGAs were available for both studies. Subgroup analyses were performed, and no relevant differences were found in sex, age (Supplemental Material Figure S1), or histology. Of interest, patients with a nonsquamous histology did not significantly benefit from anti-TROP-2 treatment compared to docetaxel (OS HR: 0.86; 95% CI, 0.73–1.01; P = 0.06; PFS HR: 0.76; 95% CI, 0.52–1.12; P = 0.17) (Figure 4a,b). However, the presence of AGAs was associated with a benefit from anti-TROP-2 therapy in terms of OS (HR: 0.63; 95% CI, 0.41–0.95; P = 0.03), but not PFS (HR: 0.59; 95% CI, 0.20–1.77; P = 0.35) (Figure 4c,d).

### 3.4. Safety

**Figure 5:**
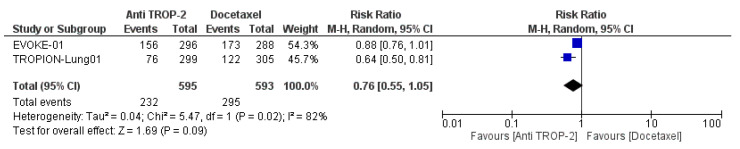
Safety of TROP-2-targeting antibody–drug conjugates vs. docetaxel in pretreated patients with advanced or metastatic NSCLC. Grade ≥ 3 treatment-related adverse events (TRAEs) were analyzed.

Compared to docetaxel, the anti-TROP-2 regimen resulted in a lower risk of developing grade ≥ 3 TRAEs (RR: 0.76; 95% CI, 0.55–1.05; P = 0.09) (Figure 5).

Notably, the two drugs exhibited slightly different safety profiles. Specifically, SG primarily produced gastrointestinal toxicity, including diarrhea and nausea, whereas 8.8% of patients treated with Dato-DXd experienced interstitial lung disease (ILD) or pneumonitis. TRAEs leading to discontinuations were less frequent with the anti-TROP-2 regimen (7.4%) compared to docetaxel (12.8%), and treatment-related deaths were rare (*n* = 7 with TROP-2-targeting ADCs and *n* = 5 with docetaxel).

## 4. Discussion

In this pooled analysis, we evaluated the outcomes of two phase III RCTs comparing the efficacy of TROP-2-targeting ADCs with docetaxel in pretreated patients with NSCLC, critically assessing the clinical utility of this treatment given the modest benefits observed in survival outcomes and its safety profile.

TROP-2 is associated, at least in the adenocarcinoma subtype, with a dismal prognosis and has been chosen as a target due to its high expression in lung tumors [25,26].

Early-phase trials showed encouraging results in patients with NSCLC, regardless of TROP-2 expression levels [16]. Despite the comparable activity between Dato-DXd and SG (both anti-TROP-2 antibodies are conjugated with a topoisomerase I inhibitor) and similar study designs, population characteristics, and sample sizes, the two trials reported discordant results [20,21].

Specifically, Dato-DXd demonstrated a significant PFS improvement compared to docetaxel (HR: 0.75; *p* = 0.004) in the TROPION-Lung01 trial, primarily driven by patients with a nonsquamous histology (HR, 0.63; 95% CI, 0.51–0.79), although no OS benefit was observed. Conversely, the EVOKE-01 study did not reveal any significant improvement of SG over docetaxel and showed no significant differences in efficacy across histologies.

According to our results, anti-TROP-2 treatment did not show a significant benefit in terms of OS or PFS over the standard of care. The significantly improved PFS observed in the reconstructed IPD analysis, which was not replicated in the inverse variance analysis, must be interpreted cautiously, considering the methodological limitations of data extraction from Kaplan–Meier curves. Furthermore, mPFS was 4.2 months with the anti-TROP-2 regimen (95% CI, 4.1–4.7) and 3.9 months (95% CI, 3.3–4.2) with docetaxel and, despite being statistically significant, this result may have limited clinical relevance.

Given the lack of OS improvement in the overall population, it is critical to further explore the potential role of TROP-2-targeting ADCs in specific subgroups, such as patients with primary or acquired resistance to anti PD-(L)1 therapies, where alternative treatment options remain limited.

In the EVOKE-01 trial, all patients received platinum-based chemotherapy and an anti-PD-(L)1-containing regimen, and a meaningful OS improvement was reported for those nonresponsive to the last anti-PD-(L)1-containing regimen. Unfortunately, this finding is not reported in the TROPION-Lung01 study, precluding a direct comparison. It is worth noting that in the TROPION-Lung01 trial, about 11% of patients had not received prior anti-PD-(L)1 therapy.

Primary resistance to ICIs is a significant challenge in NSCLC management, and TROP-2 overexpression has emerged as a potential key factor [27,28]. Analyses from the POPLAR and OAK trials showed that high TROP-2 expression correlates with worse PFS and OS of patients on atezolizumab but not chemotherapy [29,30]. This association is likely due to reduced T-cell infiltration and an immunosuppressive tumor microenvironment in TROP-2-high tumors. Plasma proteomics and multiplex immunofluorescence supported these findings, linking circulating TROP-2 levels to poor ICI outcomes. These insights highlight the potential of combining anti-TROP-2 agents with ICIs to address primary resistance and improve the outcomes of advanced NSCLC.

The most intriguing finding from our analysis is the significant OS benefit in patients with AGAs (HR: 0.63; P = 0.03; Figure 4c). In both studies, patients with AGAs received at least one prior targeted therapy. This result must be interpreted cautiously, considering that the analysis included data from only two clinical trials. Nevertheless, the observed OS benefit in patients with AGAs (HR: 0.63; P = 0.03) appears to be both statistically and clinically meaningful. According to recently proposed thresholds for a minimal clinically important difference (MCID) in survival outcomes, a hazard ratio of 0.64 corresponds to a moderate effect size (Cohen’s d = 0.5) based on the distribution-based method [31]. Given that our finding reaches this threshold, it may represent a clinically important survival benefit. However, it should be noted that the definition of MCID for hazard ratios is not yet standardized, and its application may vary depending on the clinical context, outcome type, and patient population. Moreover, this result aligns with data from the TROPION-Lung05 study [32], a phase II clinical trial that showed encouraging antitumor activity of Dato-DXd in heavily pretreated patients with NSCLC with AGAs, and was subsequently confirmed by the results from TROPION-Lung01. In the TROPION-Lung05 trial, patients with AGAs who had received at least one prior targeted therapy and platinum-based chemotherapy were included. Among these, 56.9% had an EGFR mutation, and ALK rearrangements occurred in 24.8% of patients. The study reported an overall objective response rate (ORR) of 35.8% (95% CI, 27.8–44.4), including four complete responses (total *n* = 137), and a median duration of response of 7 months (95% CI, 4.2–9.8). The safety profile was consistent with the expectations. Baseline TROP-2 tumor membrane expression, assessed by immunohistochemistry, was moderate to high in most patients, irrespective of their genomic alteration status. Notably, no significant correlation was observed between TROP-2 expression levels and the therapeutic response. Based on these results, FDA (Food and Drug Administration) accelerated approval has been requested for Dato-Dxd for patients with pretreated advanced *EGFR*-mutated NSCLC.

More recently, a phase II trial showed the encouraging antitumor activity and manageable tolerability of sacituzumab tirumotecan in previously treated patients with advanced NSCLC harboring EGFR mutations. In this study, which enrolled 64 patients with pretreated EGFR-mutant NSCLC, the ORR was 34%, with a median duration of response of 9.6 months and a mPFS of 9.3 months. Prespecified subgroup analyses based on TROP-2 expression levels showed similar ORRs between high and low expressors, but longer PFS in patients with high TROP-2 expression (ORR: 39% vs. 27%, P  =  0.306; PFS: 10.9 months vs. 8.2 months, P  =  0.100) [33].

Sacituzumab tirumotecan has recently received marketing authorization in China by the National Medical Products Administration (NMPA) for the treatment of patients with EGFR-mutant advanced NSCLC following progression on TKI therapy and platinum-based chemotherapy. This approval is based on the prespecified analysis of the OptiTROP-Lung03 study, a multicenter, randomized controlled trial evaluating the efficacy and safety of sacituzumab tirumotecan versus docetaxel in pretreated patients with EGFR-mutant advanced NSCLC. The results of this study will be presented at this year’s ASCO Annual Meeting.

Treatment with TKIs for NSCLC with AGAs has significantly improved outcomes compared to standard chemotherapy; however, the major challenge now lies in managing patients who develop treatment resistance [34,35,36]. The investigation of off-target resistance mechanisms has led to the clinical evaluation of novel therapeutic strategies, including targeted drug combinations [37,38]. The potential role of ADCs targeting TROP-2, alone or in combination, in this setting appears worth exploring. Given the encouraging data on the activity of TROP-2-targeting ADCs in pretreated NSCLC patients with AGAs, there is growing interest in exploring their efficacy in earlier lines of treatment, potentially in combination with TKIs. In this regard, we await the results of ongoing clinical trials investigating Dato-DXd in combination with osimertinib as a first-line therapy (TROPION-Lung14, NCT06350097) or following progression on osimertinib (TROPION-Lung15, NCT06417814) in patients with EGFR-mutated advanced NSCLC.

The presence of AGAs may represent a potential biomarker for selecting patients who could benefit from anti-TROP-2 therapy, either in the first-line setting or in subsequent lines of treatment. However, a key challenge remains to identify the subgroup of AGA-positive patients most likely to benefit from the addition of anti-TROP-2 agents to TKIs in the first-line setting in order to avoid the risk of overtreatment, particularly considering the increased toxicity associated with combination regimens.

In the case of EGFR-mutant NSCLC, for instance, molecular profiling to detect co-mutations such as TP53, RB1, and PIK3C, which are known to be associated with primary resistance to osimertinib [39], could help identify patients who may benefit from early treatment with anti-TROP-2 agents. Nevertheless, the urgent need remains to identify robust predictive biomarkers.

Compared to ADCs targeting HER2 or MET, which have shown significant efficacy in selected subgroups, TROP-2-targeting ADCs highlight the complexity of translating promising preclinical findings into consistent clinical benefits.

Of interest, it is known that in HER2-low or HER2-negative breast cancer, the efficacy of trastuzumab deruxtecan (T-DXd) is not primarily dependent on the internalization of the drug into tumor cells but rather on the extracellular activity of the protease cathepsin L. This enzyme, which is present in the tumor microenvironment, cleaves the T-DXd linker, facilitating payload release and inducing cytotoxicity [14].

Dato-DXd employs the same tetrapeptide-based linker as T-DXd, which is enzymatically cleavable by lysosomal enzymes such as cathepsins [40]. Therefore, extracellular payload release could also be effective in this context, potentially exerting cytotoxic effects independently of TROP-2 expression on the cell surface. A similar mechanism of action has been hypothesized for SG [41]. Specifically, the linker connecting the monoclonal antibody to SN-38 is labile, which may result in SN-38 release into the tumor microenvironment prior to internalization of the conjugate. Combined with the membrane permeability of free SN-38, this could trigger antitumor activity in neighboring tumor cells through a bystander effect.

Recent findings presented at the 2024 World Conference on Lung Cancer (WCLC) introduced the TROP-2 Normalized Membrane Ratio (NMR) as a novel predictive biomarker for TROP-2-targeting ADCs [42]. Using Quantitative Continuous Scoring (QCS), a computational pathology approach, researchers calculated the TROP-2 NMR, which reflects membrane-bound TROP-2 relative to total expression. In the TROPION-Lung01 trial, patients classified as TROP-2 QCS-NMR-positive (≥75% of tumor cells with NMR ≤ 0.56) had significantly longer PFS with Dato-DXd (median PFS: 6.9 vs. 4.1 months; HR: 0.57; P = 0.0063) compared to docetaxel. These findings underscore the potential of TROP-2 QCS-NMR as the first biomarker for ADC efficacy in NSCLC patients and highlight its promise for enhancing patient selection. However, further validation in ongoing trials, such as AVANZAR (NCT05687266) and TROPION-Lung10 (NCT06357533), is required.

Overall, anti-TROP-2 treatment demonstrated improved tolerability profiles compared to docetaxel (RR: 0.76; 95% CI, 0.55–1.05). Dato-DXd was associated with higher rates of ILD but fewer severe systemic toxicities, while SG’s gastrointestinal side effects were generally manageable with supportive care.

Future clinical trials should prioritize the exploration of TROP-2-targeting ADCs in combination with immunotherapy or novel targeted agents, leveraging their distinct mechanisms of action to enhance therapeutic efficacy in pretreated patients with NSCLC. Additionally, the role of TROP-2-targeting ADCs in patients who are nonresponsive to immunotherapy should be investigated further, as these patients represent a population with an urgent need for effective alternative therapies.

## 5. Conclusions

The results of this pooled analysis indicate that anti-TROP-2 therapy does not improve survival outcomes compared to docetaxel in previously treated patients with NSCLC, regardless of histology. However, the subgroup analysis supports the hypothesis that patients with AGAs may benefit from anti-TROP-2 treatment. We await the results of ongoing phase III randomized trials, including those evaluating combinations with TKIs, to better define the potential roles of these novel agents in this setting.

A biomarker capable of identifying patients most likely to respond is needed, given the lack of a clear correlation between TROP-2 cellular expression and drug activity, possibly due to the payload release in the tumor microenvironment.

The development of QCS-NMR represents a promising step in this direction, and we await the results of further clinical and translational analyses, such as from the ICARUS-Lung01 trial [43].

The presence of AGAs may represent a potential biomarker to identify patients more likely to benefit from anti-TROP-2 treatment. However, even in this setting, careful patient selection remains essential to determine who may truly benefit from the combination of anti-TROP-2 therapy with TKIs in the first-line setting. This approach carries the potential risk of increased toxicity, underscoring the need for a balanced evaluation of risks and benefits when considering combination strategies.

A main limitation of this pooled analysis is the inclusion of only two studies, albeit both are large, multicenter, randomized clinical trials. This constraint should be carefully considered when interpreting the findings, as it may limit the generalizability of the results and highlights the need for further evidence to support these observations.

## Figures and Tables

**Table 1:** Demographic and clinical characteristics of the patients at baseline.

		Anti-TROP-2 ADCs (*n* = 598)	Docetaxel (*n* = 609)
Age, years, median (range)		64.5 (28.5–84)	64 (28–85.5)
Sex, No. (%)	M	377 (63.01)	426 (69.95)
	F	221 (36.99)	183 (30.05)
Ethnic group, No. (%)	White	352 (58.86)	342 (56.15)
	Black	12 (0.02)	11 (0.02)
	Asian	136 (22.74)	146 (23.97)
	Other	98 (16.38)	110 (18.06)
ECOG PS, No. (%)	0	190 (31.77)	183 (30.05)
	≥1	408 (68.23)	424 (69.62)
Histology, No. (%)	Non-squamous	439 (73.41)	448 (73.56)
	Squamous	149 (24.92)	151 (24.79)
	Other	10 (0.017)	10 (0.016)
PD-L1 status	<1%	219 (36.62)	243 (39.9)
	≥1%	340 (56.86)	322 (52.87)
	Missing/Not performed	39 (6.52)	44 (7.22)
AGAs, No. (%)	Present	69 (11.53)	76 (12.71)
	Absent	529 (88.46)	533 (87.52)
Brain metastasis, No. (%)	Yes	114 (19.06)	130 (21.35)
	No	484 (80.94)	479 (78.65)
Previous lines of therapy, No. (%)	1	334 (55.85)	341 (55.99)
	2	211 (35.28)	203 (33.33)
	≥3	51 (8.53)	64 (10.51)

Note: Percentages are based on the total number of patients in each arm. The baseline is defined as the last available assessment before the start of study treatment. ADCs, antibody–drug conjugates; TROP-2, trophoblast cell-surface antigen 2; ECOG PS, Eastern Cooperative Oncology Group performance status; AGAs, actionable genomic alterations.

## Data Availability

The original contributions presented in this study are included in the article/supplementary material. Further inquiries can be directed to the corresponding author.

## Supplementary Materials

The following supporting information can be downloaded at https://www.mdpi.com/article/10.3390/cancers17111750/s1: Table S1. Summary of the authors’ judgement on the risk of bias for each selected randomized controlled clinical trial according to the Cochrane risk of bias tool for randomized trials version 2. Figure S1. Forest plot of survival outcomes based on subgroup analyses. (a, b) Overall survival (OS) and progression-free survival (PFS) by age: <65 or ≥65 years. (c, d) OS and PFS by sex.
