# Supplementary material for: Efficacy and Safety of TROP-2-Targeting Antibody–Drug Conjugate Treatment in Previously Treated Patients with Advanced Non-Small Cell Lung Cancer: A Systematic Review and Pooled Analysis of Reconstructed Patient Data
Source: Cancers (Basel). 2025 May 23;17(11):1750. doi: 10.3390/cancers17111750 (PMC12153610; doi:10.3390/cancers17111750)
Supplement: Supplementary file 1 [file cancers-17-01750-s001.zip › Table S1.pdf]

Table S1

**Unique ID**	1	**Study ID**	EVOKE-01	**Assessor**	
**Ref or Label**	doi.org/10.1200/JCO.24.00733	**Aim**	assignment to intervention (the 'intention-to-treat' effect)		
**Experimental**	Sacituzumab Govitecan	**Comparator**	Docetaxel	**Source**	Journal article(s); Trial protocol
**Outcome**	Overall survival	**Results**		**Weight**	1

Domain	Signalling question	Response	Comments
**Bias arising from the randomization process**	1a.1 Was the allocation sequence random?	Y	
	1a.2 Was the allocation sequence concealed until clusters were enrolled and assigned to interventions?	PY	
	1a.3 Did baseline differences between intervention groups suggest a problem with the randomization process?	N	
	**Risk of bias judgement**	**Low**	
**Bias arising from the timing of identification or recruitment of participants**	1b.1 Were all the individual participants identified and recruited (if appropriate) before randomization of clusters?	PY	
	1b.2 If N/PN/NI to 1b.1: Is it likely that selection of individual participants was affected by knowledge of the intervention assigned to the cluster?	NA	
	1b.3 Were there baseline imbalances that suggest differential identification or recruitment of individual participants between intervention groups?	N	
	**Risk of bias judgement**	**Low**	
**Bias due to deviations from intended interventions**	2.1a Were participants aware that they were in a trial?	Y	
	2.1b If Y/PY/NI to 2.1a: Were participants aware of their assigned intervention during the trial?	Y	
	2.2 Were carers and people delivering the interventions aware of participants' assigned intervention during the trial?	Y	
	2.3 If Y/PY/NI to 2.1b or 2.2: Were there deviations from the intended intervention that arose because of the trial context?	PN	
	2.4 If Y/PY to 2.3: Were these deviations likely to have affected the outcome?	NA	
	2.5 If Y/PY/NI to 2.4: Were these deviations from intended intervention balanced between groups?	NA	
	2.6 Was an appropriate analysis used to estimate the effect of assignment to intervention?	Y	
	2.7 If N/PN/NI to 2.6: Was there potential for a substantial impact (on the result) of the failure to analyse participants in the group to which they were randomized ?	NA	
	**Risk of bias judgement**	**Some concerns**	
**Bias due to missing outcome data**	3.1a Were data for this outcome available for all clusters that recruited participants?	Y	
	3.1b Were data for this outcome available for all, or nearly all, participants within clusters?	Y	
	3.2 If N/PN/NI to 3.1a or 3.1b: Is there evidence that the result was not biased by missing data?	NA	
	3.3 If N/PN to 3.2 Could missingness in the outcome depend on its true value?	NA	
	3.4 If Y/PY/NI to 3.3: Is it likely that missingness in the outcome depended on its true value?	NA	
	**Risk of bias judgement**	**Low**	
**Bias in measurement of the outcome**	4.1 Was the method of measuring the outcome inappropriate?	N	
	4.2 Could measurement or ascertainment of the outcome have differed between intervention groups?	N	
	4.3a If N/PN/NI to 4.1 and 4.2: Were outcome assessors aware that a trial was taking place?	Y	
	4.3b If Y/PY/NI to 4.3a: Were outcome assessors aware of the intervention received by study participants?	Y	
	4.4 If Y/PY/NI to 4.3b: Could assessment of the outcome have been influenced by knowledge of intervention received?	N	
	4.5 If Y/PY/NI to 4.4: Is it likely that assessment of the outcome was influenced by knowledge of intervention received?	NA	
	**Risk of bias judgement**	**Low**	
**Bias in selection of the reported result**	5.1 Were the data that produced this result analysed in accordance with a pre-specified analysis plan that was finalized before unblinded outcome data were available for analysis?	Y	
	5.2 ... multiple eligible outcome measurements (e.g. scales, definitions, time points) within the outcome domain?	N	
	5.3 ... multiple eligible analyses of the data?	N	
	**Risk of bias judgement**	**Low**	
**Overall bias**	**Risk of bias judgement**	**Low**	

**Unique ID**	2	**Study ID**	TROPION-Lung01	**Assessor**	
**Ref or Label**	doi.org/10.1200/JCO-24-01544	**Aim**	assignment to intervention (the 'intention-to-treat' effect)		
**Experimental**	Datopotamab Deruxtecan	**Comparator**	Docetaxel	**Source**	Journal article(s); Trial protocol
**Outcome**	Overall survival and progression-free survival	**Results**		**Weight**	1

Domain	Signalling question	Response	Comments
**Bias arising from the randomization process**	1a.1 Was the allocation sequence random?	Y	
	1a.2 Was the allocation sequence concealed until clusters were enrolled and assigned to interventions?	PY	
	1a.3 Did baseline differences between intervention groups suggest a problem with the randomization process?	N	
	**Risk of bias judgement**	**Low**	
**Bias arising from the timing of identification or recruitment of participants**	1b.1 Were all the individual participants identified and recruited (if appropriate) before randomization of clusters?	PY	
	1b.2 If N/PN/NI to 1b.1: Is it likely that selection of individual participants was affected by knowledge of the intervention assigned to the cluster?	NA	
	1b.3 Were there baseline imbalances that suggest differential identification or recruitment of individual participants between intervention groups?	N	
	**Risk of bias judgement**	**Low**	
**Bias due to deviations from intended interventions**	2.1a Were participants aware that they were in a trial?	Y	
	2.1b If Y/PY/NI to 2.1a: Were participants aware of their assigned intervention during the trial?	Y	
	2.2 Were carers and people delivering the interventions aware of participants' assigned intervention during the trial?	Y	
	2.3 If Y/PY/NI to 2.1b or 2.2: Were there deviations from the intended intervention that arose because of the trial context?	PN	
	2.4 If Y/PY to 2.3: Were these deviations likely to have affected the outcome?	NA	
	2.5 If Y/PY/NI to 2.4: Were these deviations from intended intervention balanced between groups?	NA	
	2.6 Was an appropriate analysis used to estimate the effect of assignment to intervention?	Y	
	2.7 If N/PN/NI to 2.6: Was there potential for a substantial impact (on the result) of the failure to analyse participants in the group to which they were randomized ?	NA	
	**Risk of bias judgement**	**Some concerns**	
**Bias due to missing outcome data**	3.1a Were data for this outcome available for all clusters that recruited participants?	Y	
	3.1b Were data for this outcome available for all, or nearly all, participants within clusters?	Y	
	3.2 If N/PN/NI to 3.1a or 3.1b: Is there evidence that the result was not biased by missing data?	NA	
	3.3 If N/PN to 3.2 Could missingness in the outcome depend on its true value?	NA	
	3.4 If Y/PY/NI to 3.3: Is it likely that missingness in the outcome depended on its true value?	NA	
	**Risk of bias judgement**	**Low**	
**Bias in measurement of the outcome**	4.1 Was the method of measuring the outcome inappropriate?	N	
	4.2 Could measurement or ascertainment of the outcome have differed between intervention groups?	N	
	4.3a If N/PN/NI to 4.1 and 4.2: Were outcome assessors aware that a trial was taking place?	Y	
	4.3b If Y/PY/NI to 4.3a: Were outcome assessors aware of the intervention received by study participants?	N	
	4.4 If Y/PY/NI to 4.3b: Could assessment of the outcome have been influenced by knowledge of intervention received?	NA	
	4.5 If Y/PY/NI to 4.4: Is it likely that assessment of the outcome was influenced by knowledge of intervention received?	NA	
	**Risk of bias judgement**	**Low**	
**Bias in selection of the reported result**	5.1 Were the data that produced this result analysed in accordance with a pre-specified analysis plan that was finalized before unblinded outcome data were available for analysis?	Y	
	5.2 ... multiple eligible outcome measurements (e.g. scales, definitions, time points) within the outcome domain?	N	
	5.3 ... multiple eligible analyses of the data?	N	
	**Risk of bias judgement**	**Low**	
**Overall bias**	**Risk of bias judgement**	**Low**	
