# Supplementary material for: Efficacy and Safety of TROP-2-Targeting Antibody–Drug Conjugate Treatment in Previously Treated Patients with Advanced Non-Small Cell Lung Cancer: A Systematic Review and Pooled Analysis of Reconstructed Patient Data
Source: Cancers (Basel). 2025 May 23;17(11):1750. doi: 10.3390/cancers17111750 (PMC12153610; doi:10.3390/cancers17111750)

Figure S1

(a) Overall survival by age

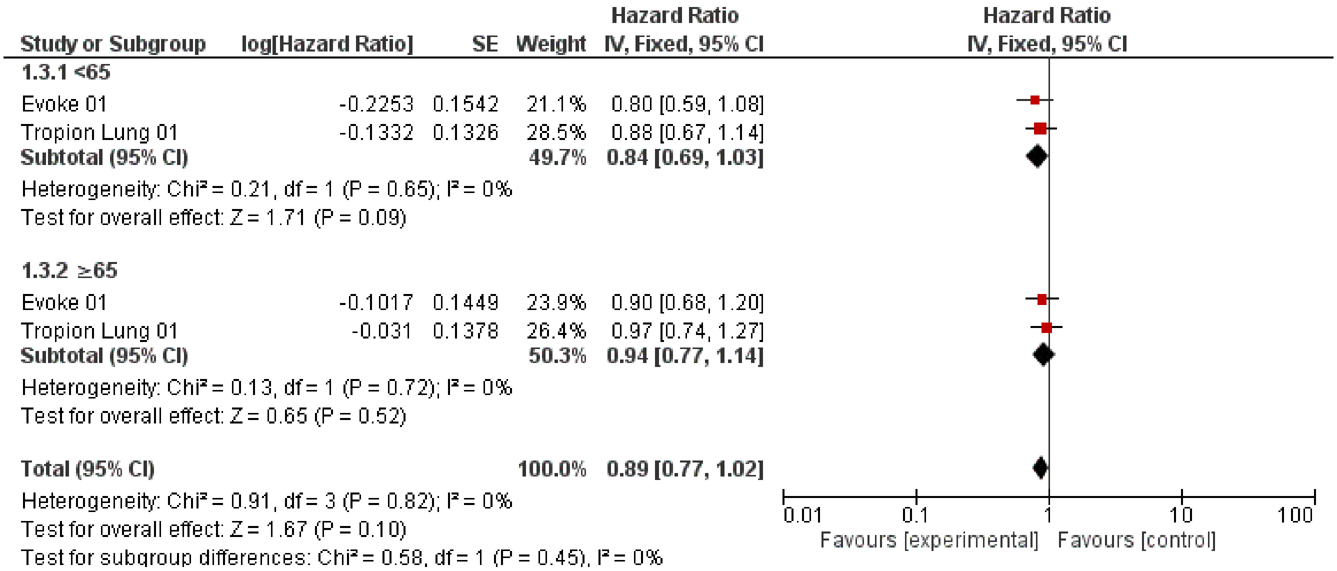

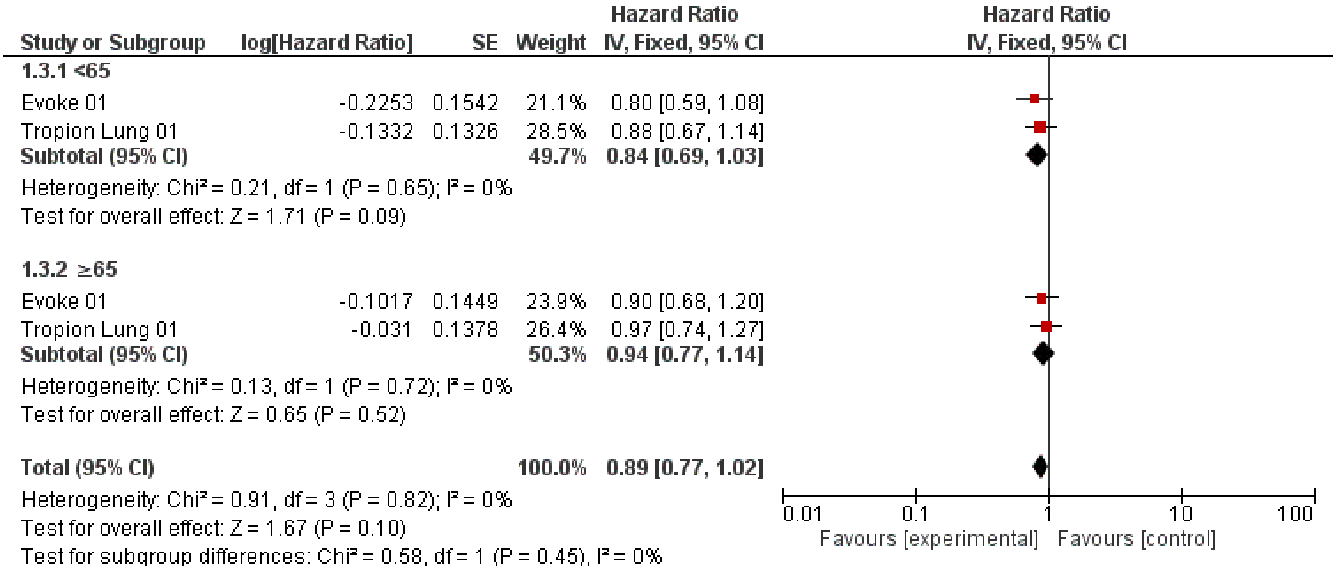

(b) Progression-free survival by age

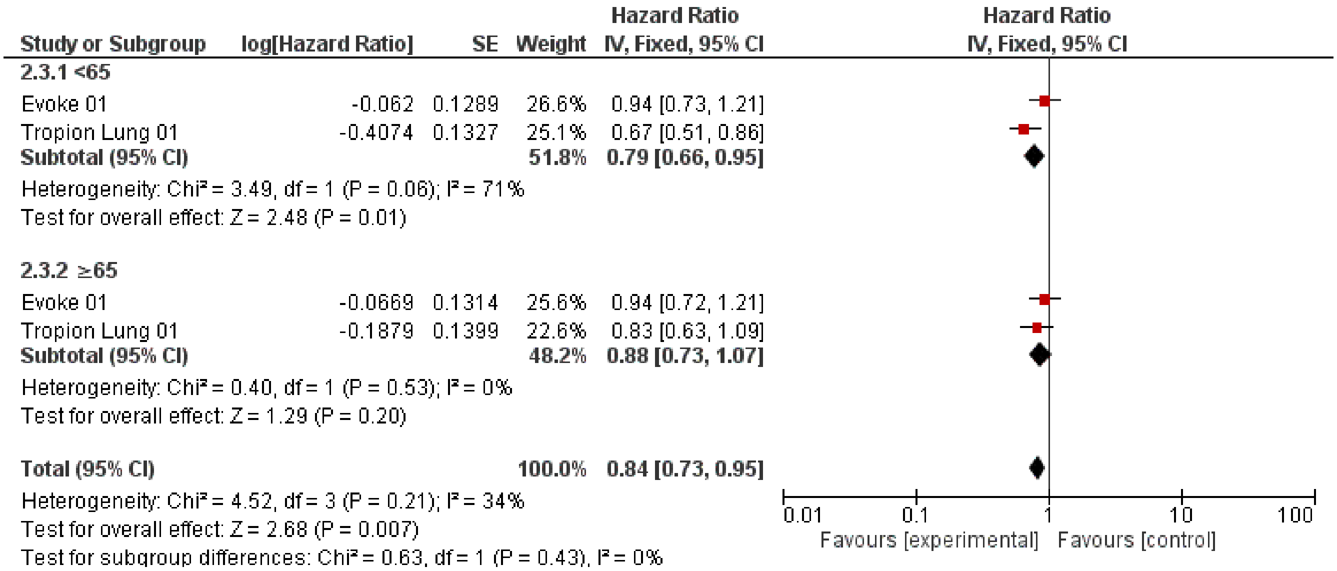

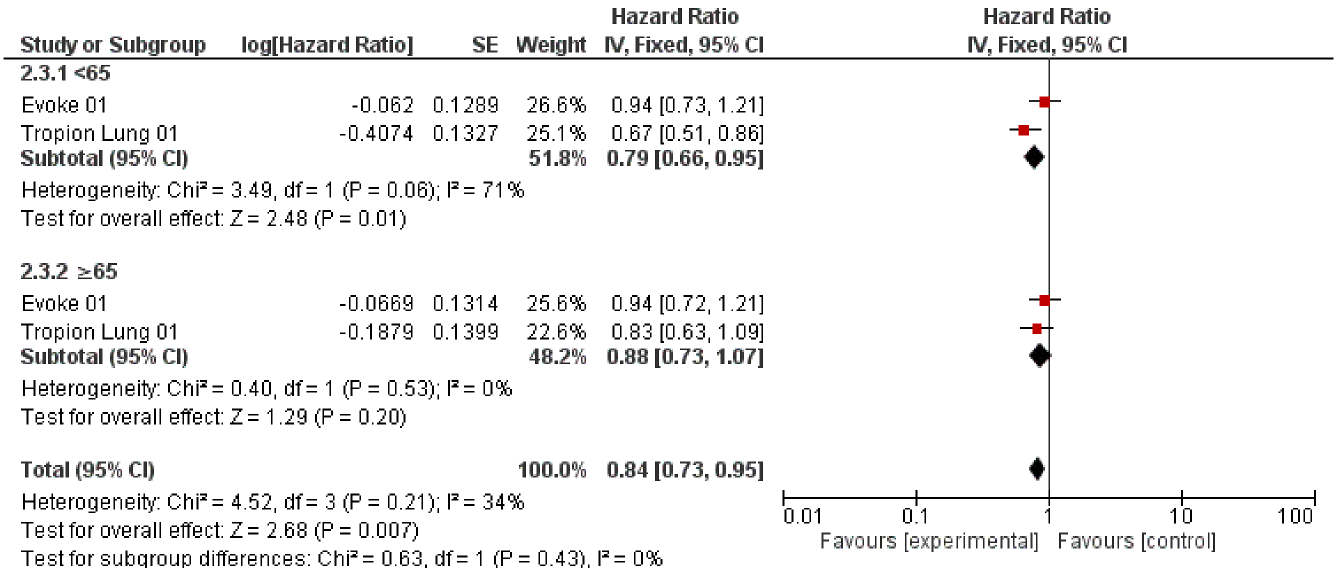

(c) Overall survival by sex

Study or Subgroup	log[Hazard Ratio]	SE	Weight	Hazard Ratio IV, Fixed, 95% CI	Hazard Ratio IV, Fixed, 95% CI
**1.4.1 Male**					
Evoke 01	-0.1128	0.1244	32.0%	0.89 [0.70, 1.14]	
Tropion Lung 01	-0.0763	0.1147	37.7%	0.93 [0.74, 1.16]	
**Subtotal (95% CI)**			**69.7%**	**0.91 [0.77, 1.07]**	
Heterogeneity: Chi² = 0.05, df = 1 (P = 0.83); I² = 0%					
Test for overall effect: Z = 1.10 (P = 0.27)					
**1.4.2 Female**					
Evoke 01	-0.1568	0.1979	12.7%	0.85 [0.58, 1.26]	
Tropion Lung 01	-0.0283	0.1675	17.7%	0.97 [0.70, 1.35]	
**Subtotal (95% CI)**			**30.3%**	**0.92 [0.72, 1.18]**	
Heterogeneity: Chi² = 0.25, df = 1 (P = 0.62); I² = 0%					
Test for overall effect: Z = 0.64 (P = 0.52)					
**Total (95% CI)**			**100.0%**	**0.91 [0.80, 1.05]**	
Heterogeneity: Chi² = 0.30, df = 3 (P = 0.96); I² = 0%					
Test for overall effect: Z = 1.27 (P = 0.20)					
Test for subgroup differences: Chi² = 0.01, df = 1 (P = 0.94), I² = 0%					

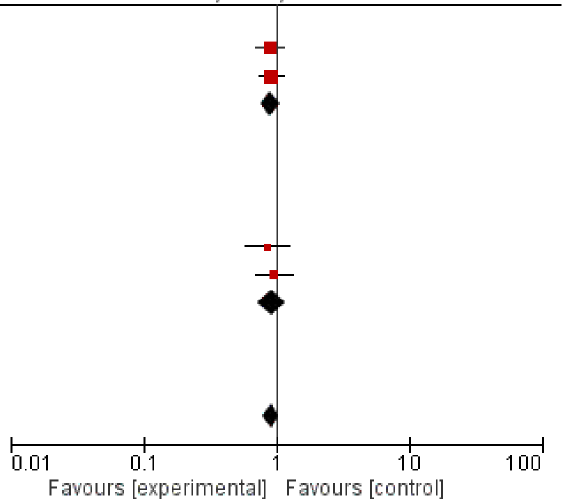

Study or Subgroup	log[Hazard Ratio]	SE	Weight	Hazard Ratio IV, Fixed, 95% CI	Hazard Ratio IV, Fixed, 95% CI
**1.4.1 Male**					
Evoke 01	-0.1128	0.1244	32.0%	0.89 [0.70, 1.14]	
Tropion Lung 01	-0.0763	0.1147	37.7%	0.93 [0.74, 1.16]	
**Subtotal (95% CI)**			**69.7%**	**0.91 [0.77, 1.07]**	
Heterogeneity: Chi² = 0.05, df = 1 (P = 0.83); I² = 0%					
Test for overall effect: Z = 1.10 (P = 0.27)					
**1.4.2 Female**					
Evoke 01	-0.1568	0.1979	12.7%	0.85 [0.58, 1.26]	
Tropion Lung 01	-0.0283	0.1675	17.7%	0.97 [0.70, 1.35]	
**Subtotal (95% CI)**			**30.3%**	**0.92 [0.72, 1.18]**	
Heterogeneity: Chi² = 0.25, df = 1 (P = 0.62); I² = 0%					
Test for overall effect: Z = 0.64 (P = 0.52)					
**Total (95% CI)**			**100.0%**	**0.91 [0.80, 1.05]**	
Heterogeneity: Chi² = 0.30, df = 3 (P = 0.96); I² = 0%					
Test for overall effect: Z = 1.27 (P = 0.20)					
Test for subgroup differences: Chi² = 0.01, df = 1 (P = 0.94), I² = 0%					

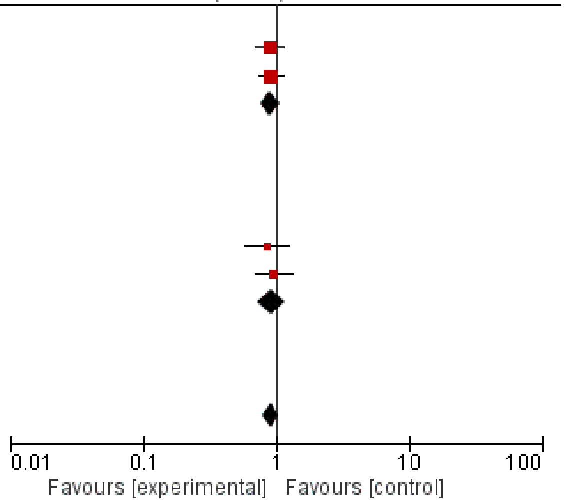

(d) Progression-free survival by sex

Study or Subgroup	log[Hazard Ratio]	SE	Weight	Hazard Ratio IV, Fixed, 95% CI
**2.4.1 Male**				
Evoke 01	-0.0437	0.1111	36.5%	0.96 [0.77, 1.19]
Tropion Lung 01	-0.2408	0.1177	32.5%	0.79 [0.62, 0.99]
**Subtotal (95% CI)**			**69.1%**	**0.87 [0.74, 1.02]**
Heterogeneity: Chi² = 1.48, df = 1 (P = 0.22); I² = 33%				
Test for overall effect: Z = 1.69 (P = 0.09)				
**2.4.2 Female**				
Evoke 01	-0.0959	0.1709	15.4%	0.91 [0.65, 1.27]
Tropion Lung 01	-0.3493	0.1705	15.5%	0.71 [0.50, 0.98]
**Subtotal (95% CI)**			**30.9%**	**0.80 [0.63, 1.01]**
Heterogeneity: Chi² = 1.10, df = 1 (P = 0.29); I² = 9%				
Test for overall effect: Z = 1.85 (P = 0.06)				
**Total (95% CI)**			**100.0%**	**0.85 [0.74, 0.97]**
Heterogeneity: Chi² = 2.94, df = 3 (P = 0.40); I² = 0%				
Test for overall effect: Z = 2.43 (P = 0.02)				
Test for subgroup differences: Chi² = 0.35, df = 1 (P = 0.55), I² = 0%				

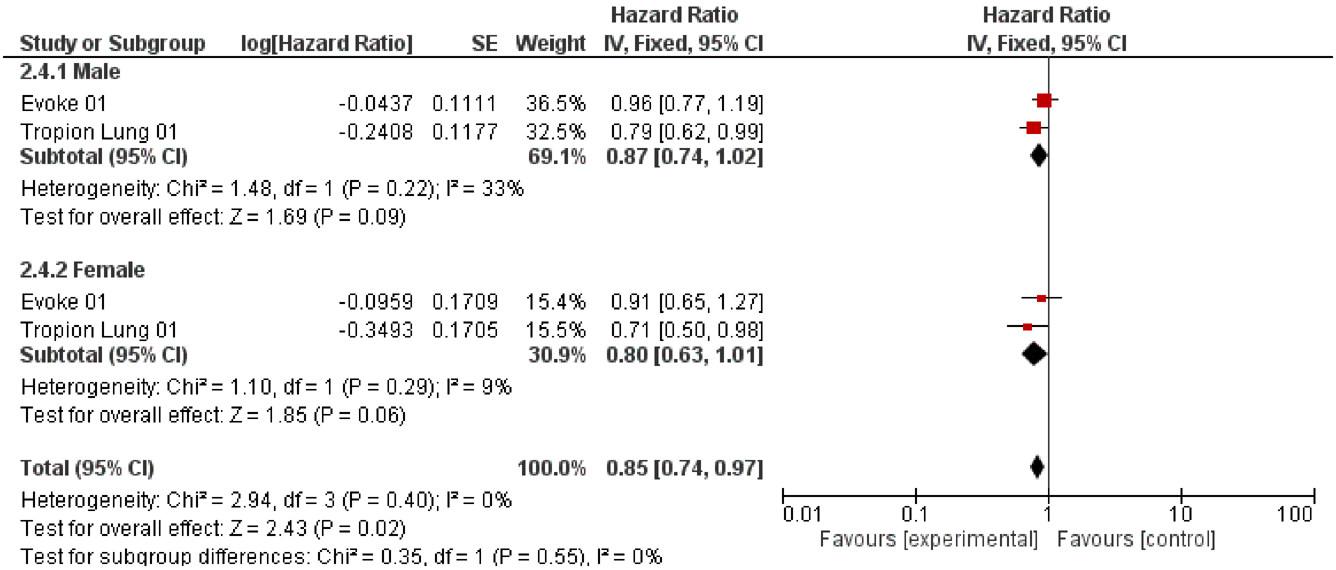

Study or Subgroup	log[Hazard Ratio]	SE	Weight	Hazard Ratio IV, Fixed, 95% CI
**2.4.1 Male**				
Evoke 01	-0.0437	0.1111	36.5%	0.96 [0.77, 1.19]
Tropion Lung 01	-0.2408	0.1177	32.5%	0.79 [0.62, 0.99]
**Subtotal (95% CI)**			**69.1%**	**0.87 [0.74, 1.02]**
Heterogeneity: Chi² = 1.48, df = 1 (P = 0.22); I² = 33%				
Test for overall effect: Z = 1.69 (P = 0.09)				
**2.4.2 Female**				
Evoke 01	-0.0959	0.1709	15.4%	0.91 [0.65, 1.27]
Tropion Lung 01	-0.3493	0.1705	15.5%	0.71 [0.50, 0.98]
**Subtotal (95% CI)**			**30.9%**	**0.80 [0.63, 1.01]**
Heterogeneity: Chi² = 1.10, df = 1 (P = 0.29); I² = 9%				
Test for overall effect: Z = 1.85 (P = 0.06)				
**Total (95% CI)**			**100.0%**	**0.85 [0.74, 0.97]**
Heterogeneity: Chi² = 2.94, df = 3 (P = 0.40); I² = 0%				
Test for overall effect: Z = 2.43 (P = 0.02)				
Test for subgroup differences: Chi² = 0.35, df = 1 (P = 0.55), I² = 0%				

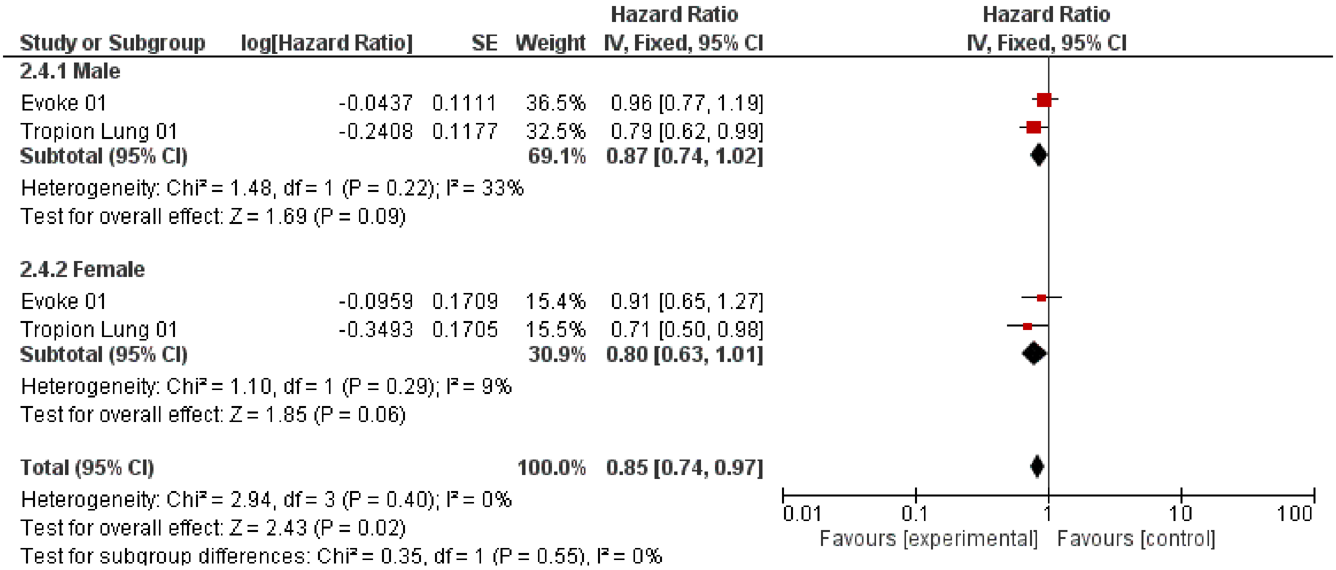

Supplement: Supplementary file 1 [file cancers-17-01750-s001.zip › Figure S1.pdf]
